# Gallantry of a British Surgeon in the Battle of Inkermann

**Published:** 1855-03

**Authors:** 


Gallantry of a British Surgeon in the Battle of Inkermann.—A correspondent of the *Times*, in the Crimea, writes: "At one time, while the Duke of Cambridge was rallying his men, a body of Russians began to single him out, and to take shots at him in the most deliberate manner. A surgeon of a cavalry regiment, Dr. Wilson, 7th Hussars, who was attached to the brigade, perceived the danger of His Royal Highness, and with the greatest gallantry and coolness assembled a few men of the Guards, led them to the charge, and utterly routed and dispersed the Russians. The Duke's horse was killed in the course of the fight. At the close of the day he called Dr. Wilson in front of the regiment, and publicly thanked him for having, in all probability, saved his life.

